# A call to action for adverse drug event (ADE) detection and prevention

**DOI:** 10.3389/fdgth.2025.1507967

**Published:** 2025-03-28

**Authors:** John McCue, C. David Butler, Raymond C. Love, Shelly Spiro, Roy Guharoy, the United States Pharmacopeia (USP) Allergy & Intolerance Classification Expert Panel

**Affiliations:** ^1^ Healthcare Quality and Safety Center of Excellence, United States Pharmacopeia, Rockville, MD, United States; ^2^ Curatro, Richmond, VA, United States; ^3^ School of Pharmacy, University of Maryland, College Park, MD, United States; ^4^ Pharmacy Health Information Technology (PHIT) Collaborative, Alexandria, VA, United States; ^5^ University of Massachusetts Medical School, Worcester, MA, United States

**Keywords:** allergy, intolerance, patient history, healthcare, standardization

## Abstract

Injury from medication use, known as an adverse drug event (ADE) accounts for millions of emergency department visits globally and thousands of hospitalizations annually within the United States. Efforts to prevent and detect ADEs within healthcare systems are complicated by data quality, lack of data standardization, and actionable clinical decision support systems. United States Pharmacopeia (USP) proposes the use of an ADE value set, a standardized grouping of medical terms, to improve the identification, documentation, and use of ADE information in EHRs. Artificial Intelligence and Machine Learning capabilities would be further strengthened through the standardization of ADE data and information.

## Introduction

The inability to detect and prevent injuries from medication use, also known as adverse drug events (ADEs), results in approximately 1.5 million emergency department (ED) visits and almost 500,000 hospitalizations each year (1). Based on 96, 925 cases analyzed from 2017 to 2019 (mean patient age, 49 years; 55% female), there were an estimated 6.1 (95% CI, 4.8–7.5) ED visits for medication harms per 1,000 population annually and 38.6% (95% CI, 35.2%–41.9%) resulted in hospitalization (2). ADE detection and prevention within healthcare settings heavily relies on information within an electronic health record (EHR) to provide information that informs clinical decisions. Efforts to improve ADE prevention by capturing both active and inactive ingredients within EHR data is underway but are incomplete and met with competing approaches. Additionally inactive ingredients, also referred to as excipients, are increasingly causing an allergic reaction even though their inclusion in a drug product is to facilitate absorption or improve stability, taste, or appearance (3).

Many factors lead to the inaccurate or incomplete capture of medication information that informs ADE prevention and detection. Varying terminology and a lack of consistent use by both practitioners and IT systems for all ingredients contained in a medication product formulation can lead to inconsistent use of active ingredients, inactive ingredients, therapeutic class names, generic names, chemical names, and trade names within ADE documentation (4). The power of ADE detection and prevention in an EHR comes when ADE information can be used by clinical decision support systems (CDSS), but current practices are costly and time-consuming. A study by McCoy (5) reported clinicians reviewing 56 total CDSS alerts per day with an average time of 49 min per day processing those alerts. CDSSs do not currently incorporate and relate ADE information to other factors in the patient's medical record in a manner that allows easily automated prediction of potential (or recurrent) ADEs prior to manifestation. Nor are current CDSSs designed to recommend methods to mitigate an ADE. As this content is technical and incorporates abbreviations used within health information and technology, please refer to Table 1 for acronyms used throughout this paper.

**Table 1:** Acronyms used throughout this paper.

Acronym	Explanation
ADE	Adverse Drug Event
AI	Artificial Intelligence
C-CDA	Consolidated Clinical Documentation Architecture
CDSS	Clinical Decision Support System
CI	Confidence Interval
ED	Emergency Department
EHR	Electronic Health Record
FDA	U.S. Food and Drug Administration
FHIR	Fast Healthcare Interoperability Resources
HL7	Health Level 7
ICD	International Classification of Diseases
IT	Information Technology
LOINC	Longitudinal Observation Identifiers Names and Codes
ML	Machine Learning
ONC	Office of the National Coordinator for Health IT
RXCUI	RxNorm Concept Unique Identifier
RxNorm	U.S. Standardized Naming System for Medications
SNOMED CT	Systematized Nomenclature of Medicine Clinical Terms
USCDI	United States Core Data Interoperability
USP	United States Pharmacopeia

## Solution

For ADE information to travel, a common language or codified language is needed. The codification of health diagnoses has been established by International Classification of Disease version 10 (ICD-10) codes (6) and United States Core Data Interoperability (USCDI) codes (7). While these terminologies have been integral to making health diagnoses information transferrable and interoperable, they have limitations in associating complete information related to medications and ADEs, as medications and ADEs utilize varying terminologies used within healthcare data. This directly results in decreased actionability of ADE detection and prevention within EHRs. ADE information will need to apply a set of standardized and grouped codes so that information can be documented, stored, transferred, interpreted, and made actionable irrespective of the pharmacy and/or EHR platform used.

To identify a solution to prevent more ADEs, United States Pharmacopeia (USP) convened a panel of experts. Through extensive literature review, system analyses, and expert discussion, the panel identified solutions for standardization and interoperability of ADE information.

To produce an ADE solution, the expert panel has identified that it is important to establish a common set of standards for ADE definitions and capture. Standardized ADE data capture and hierarchy can enable an interoperable dataset that can follow a patient to other EHR systems throughout that patient's life. While standardization of information heavily relies on accurate data capture from healthcare provider documentation, it also relies on standardization of data and healthcare data terminology. One solution to enhance terminology standardization and interoperability is the use of a data value set, which is a list of specific values, terms, and their codes, used to describe clinical and administrative concepts in quality measures. Value sets provide groupings of unique values along with a standard description or definition from one or more standard vocabularies used to describe the same clinical concept (8). For example, value sets can be used within healthcare to standardize clinical terms related to ACE-inhibitors or other medications throughout different terminologies (9). The development of a value set addressing ADEs can standardize data entry and recording for use in CDSS and facilitate interoperability across EHR, pharmacy, clinical, and other health platforms.

In creating a value set, the expert panel's strategy is to model and enhance standard ADE documentation by electronically mapping drug products with common clinical manifestations, with an end goal to improve and protect patient safety. By electronically mapping drug products to clinical manifestations, clinicians will have more accurate information that can establish whether a clinical manifestation is likely to occur due to a specific chemical entity (active ingredient vs. excipient). The value set also features standardized, interoperable codes to improve clinical decision making; interoperability of patient ADE information across EHRs; effectiveness and quality of alerts; and reduction of excessive drug alerts by minimizing repeat reactions in patients with an ADE value set tool.

The value set is designed to address 4 specific aims:

1. To utilize standard terminology, allowing an exchange of coded sets across multiple platforms and settings (interoperability) that will ensure ADE information is accessible and visible to all healthcare practitioners for treatment decision making.
2. To utilize a value set to expand ADE documentation to include both accurate, complete drug product information and the clinical manifestation in standardized language attached to terminology.
3. To add a layer of medication safety through a more targeted clinical decision support message for the clinician on possible future ADEs based on a patient's current medical history.
4. To improve data collection and information that informs the frequency of ADEs and reveals previously undocumented ADEs.

The Expert Panel recommends two models for utilization of the value sets: Fast Healthcare Interoperability Resources (FHIR) and Consolidated Clinical Documentation Architecture (C-CDA). These models are based on the Health Level 7 (HL7) Allergy Domain Analysis Model (10) and were required by the United States Office of the National Coordinator for Health IT (ONC) for meaningful use in certified EHRs. C-CDA is a popular, flexible markup standard developed by HL7 that defines the structure of certain medical records, such as discharge summaries and progress notes, to ensure efficient exchange of this information between providers and patients (11). FHIR is an interoperability standard for electronic exchange of healthcare information (11). These models support key elements of a complete ADE Observation documentation. The key elements include the culprit substance [encoded using RxNorm Concept Unique Identifier (RXCUI)], clinical manifestation [encoded using Systematized Nomenclature of Medicine Clinical Terms (SNOMED CT)], severity, criticality, and type/category of reaction.

Interoperability can be achieved by formatting in a way to model the index and prospective drug pairs with their shared clinical manifestation. Index and prospective drugs will be mapped to RxNorm and will utilize FDA databases where applicable. The clinical manifestations will map to SNOMED CT, which has been proposed for use with the Quality Data Model as a “medication class” vocabulary option (12).

**Figure 1:**
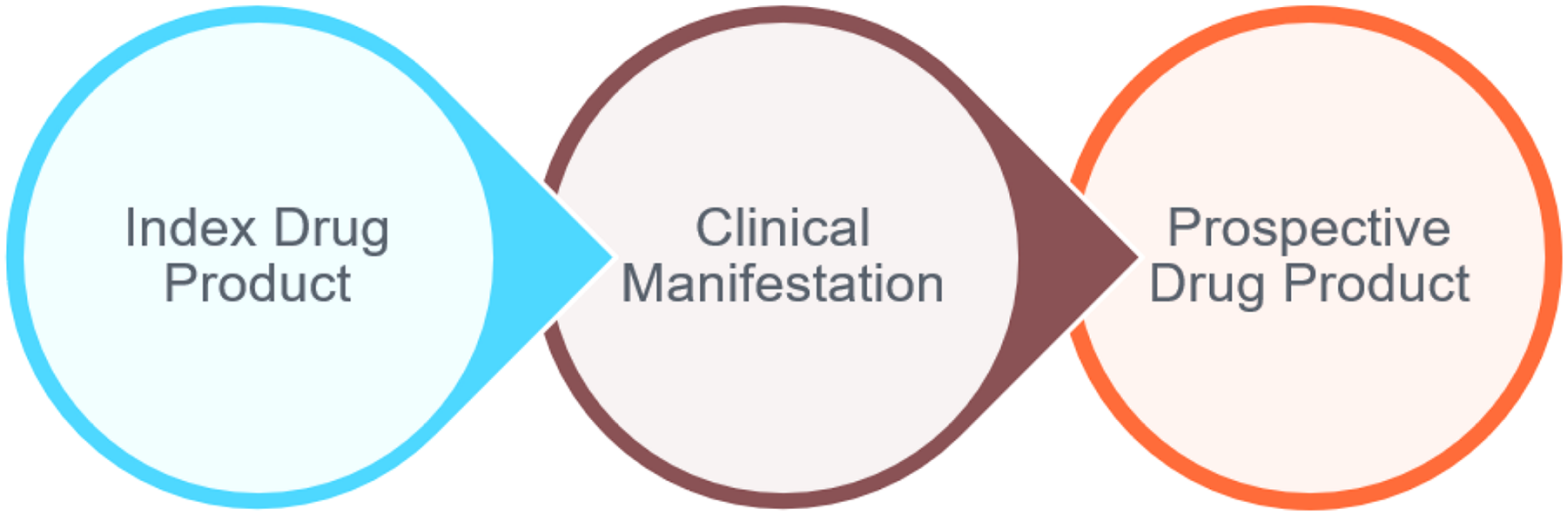
Allergies & intolerance value set structure.

By establishing a standard terminology for each component, the value set will establish a universal coded language for these terms. The value set will be structured in a way that will allow the drug product (or specific chemical when identified) that caused the clinical manifestation (e.g., index drug product) to be cross-referenced with the prospective drug product and result in a clinical decision support message to the clinician on the potential risk of the reaction re-occurring. An illustration of the ADE value set mapping is detailed in Figure 1.

While an ADE value set brings forward much potential, there are known challenges in creating, maintaining, and implementing a value set. Value set creation is time consuming and requires a continuous manual process to identify, create, and maintain mapping of data elements. For the value set to be effective, data must be appropriately mapped from different data standards and formats, including FHIR, HL7, USCDI, C-CDA, SNOMED CT, RXNORM, and Longitudinal Observation Identifiers Names and Codes (LOINC). If mapping is completed incorrectly, value set functionality will be impaired or inaccurate. Data elements from different terminologies may also have challenges in being mapped to each other. The creation of these value sets also requires considerable stakeholder engagement throughout the health informatics ecosystem, including terminologists, EHR vendors, informaticists, clinicians, and other key stakeholders. Barriers to implementing a value set include healthcare stakeholder consensus on how to implement, healthcare consensus on standardized terms and application of medication data elements, regulatory barriers, financial barriers, and clinician education. Increased adoption of new technologies, such as machine learning (ML) and artificial intelligence (AI), have the potential to aid in these challenges and make these processes less burdensome.

## Conclusion

USP aims to initiate the development, refinement, and use of an ADE Value Set to improve and clarify documentation of adverse drug reactions, drug allergies, drug side effects, and other drug intolerances. This value set tool will improve the identification, documentation, and use of patient ADE information in EHRs. Such a resource would reduce the risk of repeat reactions in patients who have history of an ADE, as well as prevent the onset of a new ADE for a patient with demographic and genomic preclusions to a possible ADE, by clarifying their medical history, making it more meaningful to clinicians.

USP calls on other pertinent organizations across the healthcare industry to work together on creating an interoperable system incorporating all pertinent drug product information, monitoring, and analyzing methodologies to be used by practitioners and IT systems, appropriately implementing financial and cost-assessment policies and procedures, and researching further refinement of ADE and drug product database repositories to be made available for the detection and prevention of ADEs. If EHRs incorporate the proposed value set into their systems, clinicians may be presented with fewer but more accurate, critical alerts which influence clinical decision-making in a way that minimizes risk of an ADE. This value set would be available industry-wide and has the potential to consolidate available data, make information more accurate, and allow CDSSs to better inform providers and prescribers providing patient care.

An ADE value set has the potential to be scaled for additional use cases and additional functionalities. One potential future functionality is applying pharmacogenomic data to an ADE value set, allowing healthcare practitioners to weigh a patient's predisposition to an ADE based on genomic variants. Acknowledgement of patient variability and the implications of pharmacogenomics are crucial to the understanding and management of a patient's ADE and to safe medication-prescribing practices. Additionally, as AI concepts continue to mature, there may be AI capabilities that can be used to increase use and functionality of ADE value sets to incorporate even more patient and drug product parameters, such as renal function, liver function, or medication information generated from other sources, such as health information networks, health information exchanges, or other electronic sources.
